# Tradeoffs between pace and reach of patient decision aids: a case study adapting a breast reconstruction decision aid from clinical implementation to social media dissemination

**DOI:** 10.1186/s43058-026-01062-2

**Published:** 2026-07-25

**Authors:** Ana A. Baumann, Clara N. Lee, Krista Cooksey, Viktoria Vonder Haar, Kathryn Chetwynd, Victor Catalan Gallegos, Victoria Triana, Lisa Carter-Bawa, Eva A. May, Vivian Muzyk, Mary C. Politi

**Affiliations:** 1 Division of Public Health Sciences, Department of Surgery, Washington University School of Medicine, St. Louis, MO USA; 2 Division of Plastic and Reconstructive Surgery, University of North Carolina, Chapel Hill, NC USA; 3 Bursky School of Public Health, Washington University in St. Louis, St. Louis, MO 63130 USA; 4 Hackensack Meridian Health Center for Discovery & Innovation, Nutley, NJ USA; 5 Georgetown Lombardi Comprehensive Cancer Center, Georgetown University, Washington, DC USA; 6 UNC Lineberger Comprehensive Cancer Center, Community Advisory Board, BREASTChoice Study, Chapel Hill, NC USA; 7 UNC Lineberger Comprehensive Cancer Center, Patient and Community Engagement to Educate Researchers (PEER) Advisory Committee, Chapel Hill, NC USA

**Keywords:** Patient decision aids, Dissemination, Implementation, Social media, Electronic health record

## Abstract

**Background:**

Patient decision aids (PtDAs) support shared decision making and can improve patient outcomes. Nevertheless, PtDAs have not been widely adopted in healthcare settings because of numerous patient-, clinician-, and organizational-level factors. To address the slow pace of implementation and limited reach of PtDAs, we adapted a PtDA from implementation through the electronic health record in clinical settings for dissemination on social media and online communities.

**Methods:**

We describe the adaptation of BREASTChoice, a PtDA about breast reconstruction after mastectomy, originally used in clinical settings and the electronic health record (EHR), for dissemination through social media and online communities. We conducted focus groups and interviews with patients and topical experts to guide the adaptation process, using Rogers’ Diffusion of Innovations and ADAPT-ITT, and tracked adaptations using FRAME.

**Findings:**

Based on our findings, we propose a model that identifies differences in accelerators and inhibitors of BREASTChoice and its implementation and dissemination strategies, with the goal of outlining how speed of implementation and dissemination can differ between contexts.

**Implications for dissemination and implementation research:**

This study provides a model that explicitly outlines the potential determinants of implementation and dissemination of PtDAs both within and outside of health settings. Evaluating these determinants will help researchers and PtDA developers to increase speed and reach of PtDAs while maintaining scientific rigor.

Contributions to the literature

- Speed remains an understudied construct in dissemination and implementation sciences. This study compares the pace and reach of EHR-based implementation with dissemination through social media and online communities.
- While there is a moral imperative to increase the pace of implementation and broaden the reach of PtDAs, we propose first slowly and deliberately adapting PtDAs to preserve rigor, usability, and increase sustainability potential.
- We elucidate a model that identifies determinants for implementation and dissemination of PtDAs to expand reach and increase the pace of reach to communities within and outside health centers.

## Background

Patient decision aids (PtDAs) are tools designed to support shared decision making (SDM) between clinicians and patients [1]. They describe information about decisions, possible options and outcomes (e.g., benefits/harms), help patients clarify the values they place on benefits and harms, and encourage a discussion about which possible outcomes matter most to a patient. PtDAs have been developed in different formats, including paper-based pamphlets, mobile phone applications, videos, electronic health record (EHR) integrated decision support with patient portal components, and interactive websites [2]. Across many contexts, PtDAs increase patients’ understanding of their conditions, reduce decisional conflict and encourage active engagement in health decisions [3, 4]. 

However, despite decades of research to develop PtDAs in numerous formats and evaluate their benefit, PtDAs have not been widely implemented into clinical practice [5]. Clinician-level barriers to routine implementation include the perception that they might increase the time of consultations, lack of training or skill in using them, lack of models for how to use them in clinical care, or lack of knowledge about their benefits [5–7]. At the organizational level, barriers to adoption include human–computer interaction issues for computer-based tools, misalignment between organizational incentives and PtDA use, and fear of disrupting clinical workflow [8]. Limited implementation or use of PtDAs could exacerbate health and healthcare inequities, whereby only those with enough knowledge, access, health literacy skills feel empowered to play an active role in their health [9, 10]. 

Social media and online communities (groups of people with shared interests who connect primarily through the internet) present an opportunity to simultaneously increase access to information in PtDAs and bypass the clinical workflow barriers to their use. Disseminating PtDAs through social media could have several benefits to patients: accessibility, low cost to users, wide reach, and ease of sharing [11, 12]. Most people in the U.S. use the internet (93%), have home broadband internet (75%), own smartphones (86%), and use social media (81%, up from 5% in 2005) [13]. Disparities in access to broadband internet are declining [14, 15]. Importantly, people are searching for health information online [12]; directing them to relevant, evidence-based, and rigorously developed PtDAs could enhance their access to quality information and involvement in important decision-making processes. Sharing PtDAs through social media could support SDM and reduce inequities in PtDA use by activating patients without relying on clinicians or hospital systems to adopt the infrastructure for clinical implementation, if PtDAs are able to reach populations who can most benefit from them.

Speed is an understudied variable in the fields of dissemination and implementation, and more work is needed to understand the variables and conditions that most likely impact speed of use of evidence-based practices [16]. The goal of our research is to examine whether we can increase the speed of implementation of PtDAs, especially to all relevant populations, by sharing and promoting them online. We posit that there could be slower speed when implementing in healthcare organizations such as through the EHR, and faster speed if shared through social media. However, speed to increase the reach of PtDAs intersects with accuracy of reach: implementing in healthcare organizations may have greater accuracy in reaching the target population, (e.g., those considering mastectomy with reconstruction), whereas sharing through social media could reach people who are not facing the decision. We are not advocating for either approach, but rather recognizing that the approaches leverage different accelerators (mechanisms of change that reduce the time to impact of an intervention) and leakages (points of breakdown or inhibitors that delay impact).

Changing an intervention from one setting (clinical) to another (social media and online communities) needs to be done with care: while there is a moral imperative to actively share effective interventions and to support patients who need the intervention the most, doing so without carefully considering how to adapt, what to adapt, and whose voices are included in the adaptation process, could pose future harm and not sustain the intervention long term. Moving PtDAs from clinical settings to social media and online communities entails adapting the intervention to better fit the new context. It also requires a change in the initiator of using a PtDA from the clinician to the patient. Adaptation is defined as *“*a process of thoughtful and deliberate alteration to the design or delivery of an intervention, with the goal of improving its fit or effectiveness in a given context*”* [17]. The science of adaptation of both interventions and strategies, and of community engagement, has been long recognized in the field of implementation science as an important aspect of supporting adoption and sustainability of evidence-based practices [17–21].

In this paper, we present a case study of the adaptation of BREASTChoice, a PtDA about breast reconstruction after mastectomy for breast cancer, originally developed for clinical use and EHR integration for dissemination on social media and online communities. Before dissemination, we invested in a deliberate adaptation process to preserve the rigor, usability, and sustainability of BREASTChoice. We describe the impact on speed and reach and discuss future work in this area.

### The BREASTChoice patient decision aid: Specific EHR related challenges and opportunities

BREASTChoice is a PtDA designed to facilitate decisions about breast reconstruction after mastectomy for breast cancer. The tool includes three components: (1) education and values clarification about the options of whether to have reconstruction, when to have it, and what type to consider, (2) a personalized risk prediction model about major complications from immediate reconstruction, using clinical data from a patient’s EHR, and (3) a clinician- and patient-facing summary page to support shared decision-making [22]. The tool was developed with extensive user input and tested in two randomized trials (a single site randomized trial and multisite hybrid/effectiveness implementation trial) [23–28]. 

Despite positive impact on patients’ decision quality and original agreement from clinical teams to build it into the EHR for broader use, after the trials ended, clinicians were unable to mobilize resources in hospital systems to maintain it [24, 28]. In addition, some surgeons did not always open or review the EHR until after the reconstruction consultation, limiting clinical implementation even if hospital systems were willing to support maintenance. From patients’ perspectives, some were concerned about privacy and data security. Others could not access the tool in advance of their appointment due to the timing of their appointments, with a referral link required to be sent from a care team member. Some patients stated that not everyone in their personal networks had access to evidence-based information about options and they wanted to share the information with their family members or supportive others. Some requested broader shareability to support conversations in their cancer support networks about their breast reconstruction decisions. Programming challenges also impacted implementation. For example, at one site, clinicians received a blank summary page, instead of one that included the patient’s personal preferences and estimated risk [22]. Finally, BREASTChoice could not reach potential users who were not connected to academic medical centers with the informatics infrastructure, resource, and costs to support implementation.

In summary, despite effectiveness in improving decisions and support from patients, clinicians, informatics experts, and hospital leaders, hosting the PtDA in an EHR entailed dependence on clinicians or hospital staff and leadership to: (1) have a conversation with patients to support SDM; (2) deploy infrastructure and funding available to support use and maintenance; (3) have an established workflow set up for EHR connection and patient portal use prior to patient encounters; and (4) have patients engaged in the patient portal to review and receive information prior to clinical encounters. These factors affected key outcomes such as patients’ decision quality, BREASTChoice sustainability, and equity in use and clinical benefit across users who did not or could not receive the tool. In other words, the pace of reaching patients who may need BREASTChoice the most (i.e. those without prior knowledge, experience or empowerment to actively engage in decisions), could be considered limited and slow when implemented through the EHR alone because the process of implementation was bounded by intervention and organizational factors [29]. 

Dissemination science has been defined as “the scientific study of the targeted distribution of information to a specific professional person or group of professionals” [30] as well as “the identification of translatable methods to increase intentionally and equitably the public health demand.” [31] Reviews of dissemination science have called for further understanding of mechanisms of action and outcomes of the dissemination process [30, 32–34]. This paper fosters the science of dissemination by proposing potential accelerators and inhibitors of reach and pace of sharing PtDAS through social media and online communities. In the case of BREASTChoice, our aim was to increase overall reach, precision of reach, and pace of reach to broader groups of patients with breast cancer to enhance their decision-making process about breast reconstruction choices. Our own and others’ research on social media-based health interventions suggest that breast cancer survivors making these decisions would likely engage with BREASTChoice in these platforms [35, 36]. 

Before we began the process of increasing the pace of reach by adapting the intervention for social media and online communities, we first slowed the process to identify the core features of BREASTChoice that should be preserved and the features that could be adapted. We engaged with patients and experts to effectively adapt the PtDA; below we describe the process of adaptation.

## Methods

### BREASTChoice: Identifying its core components

BREASTChoice needed to have the following core components of a PtDA based on international standards [37]: (1) description of the specific decision to be made [for BREASTChoice, this included information about breast reconstruction procedures, possible reconstruction types, possible timing of the procedures, and numerous and diverse photos demonstrating short- and long- term outcomes across options]; (2) guidance and support for patients to clarify personal preferences and values [for BREASTChoice, this included sections asking people to rate how they felt about attributes of options such as complication risk, recovery time, and appearance]; (3) support for discussions with medical professionals to guide health decisions that match preferences rather than focus on recommending an option [for BREASTChoice, this included a summary page of information and preferences tailored to the patients’ context]. In addition, BREASTChoice included a unique personalized risk prediction model for complications from reconstruction, developed and validated across multiple studies. These elements could not be appropriately adjusted for online dissemination without considering how to adapt the PtDA from its original design.

### Frameworks guiding the adaptation process

The approach to adaptation of BREASTChoice for online dissemination was guided by the Diffusion of Innovations Theory [38]. We considered the intervention’s attributes (e.g., BREASTChoice’s effectiveness, cultural relevance, complexity, relative advantage and evidence-base), the characteristics of the target population (e.g., breast cancer survivors’ interest in innovation, access to decision support, comfort actively engaging in care, and use of social media), and the social system (e.g., who survivors and clinicians looked to as key, trustworthy opinion leaders and perceived social pressure to adopt BREASTChoice) [38, 39]. We also considered ethical issues that would arise with broader dissemination. For example, we considered whether password protection was needed, since the tool contains real patients’ photos, which were originally intended for patient education and research, and not for broad use on social media. We ultimately removed some photos and only included those with consent given for broad social media dissemination, rather than slowing the speed and reach by requiring a password which can create barriers to access. We will continue to monitor site usage given the sensitive material.

**Fig. 1:**
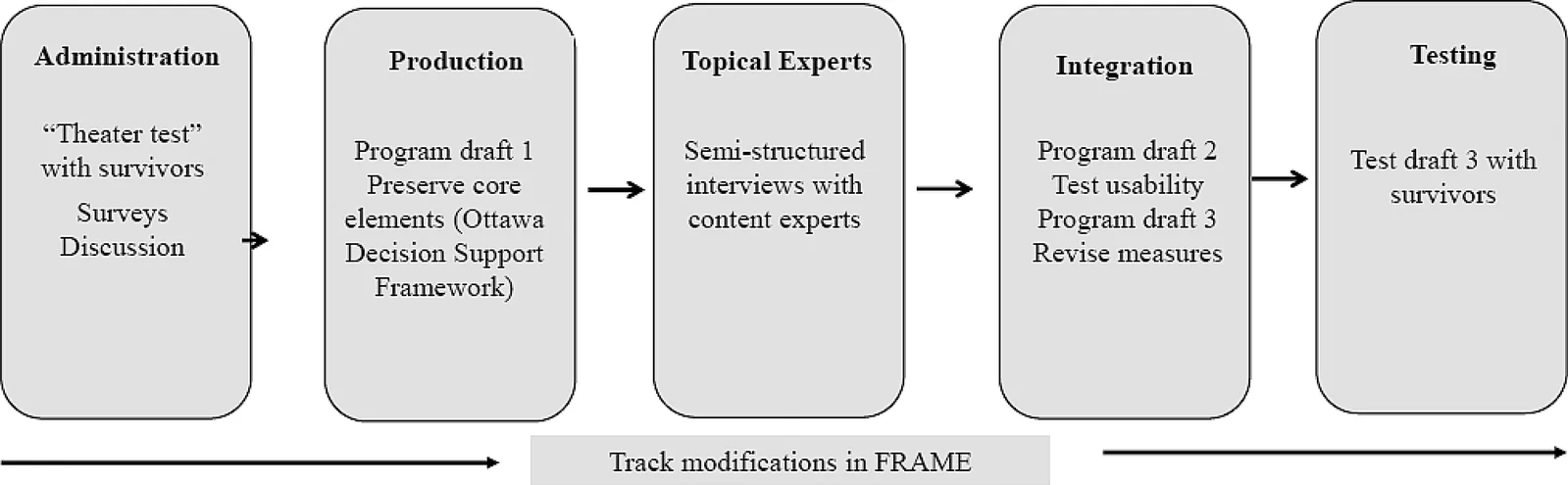
Application of ADAPT-ITT model to BREASTChoice

We used the ADAPT-ITT framework to guide the process of adaptation of the intervention itself [40]. ADAPT-ITT takes a user-centered design approach [41] to adapting an evidence-based intervention to a different setting, population, or cultural context. While ADAPT-ITT was originally used in the context of HIV prevention [40], it has been used in other contexts such as chronic disease, cardiovascular care, mental health and oncology [42]. Related to the BREASTChoice context, ADAPT-ITT has also been used to modify interventions for digital use [43–45]. We selected ADAPT-ITT for its structured, sequential approach to community and expert engagement, which aligns with recommendations on engaging with patients, health practitioners, and decision aid developers and researchers [46]. We also chose ADAPT-ITT because it emphasizes input from the target population while preserving the intervention’s core elements, and because of its relative simplicity compared to other models [40]. The framework has the following phases: assessment, decision, administration, production, input from topical experts, integration, training and testing [40]. We were attentive to the process of adaptation and included multiple voices (voices of patients with different demographics and psychographics, and of topical experts in breast cancer, social media, and/or health equity; see below) so as to explicitly define what would and would not be adapted to make BREASTChoice available, engaging and shareable through social media. Finally, the adaptations made to BREASTChoice were tracked using Framework for Reporting Adaptations and Modifications-Enhanced (FRAME), including “who, what, when, where, why, and how” the adaptations were made [17]. Suggestions that were not executed were also documented, along with the rationale for their non-implementation. Figure 1 shows our application of ADAPT-ITT model to BREASTChoice.

### Study design

We conducted focus groups and interviews with breast cancer survivors and topical experts to inform the adaptation process of BREASTChoice.

### Participants and study procedures

We conducted several meetings with our Community Advisory Board (CAB), which included 2 clinicians and 3 breast cancer survivors, to discuss potential adoption of the tool on social media and readiness for change, focus groups with breast cancer survivors (*n* = 14 across 3 focus groups), and qualitative interviews with topical experts (*n* = 9). Meetings with the CAB occurred between October-November of 2024, focus groups were conducted in January 2025, and interviews with topical experts were conducted April-May of 2025.

Focus groups were conducted with breast cancer survivors who: were assigned female at birth, could understand an Institutional Review Board-Approved Consent Information Sheet, were ≥ 18 years at the time of consent, had a history of breast malignancy or of elevated risk (e.g., BReast CAncer gene – BRCA - mutation) for breast malignancy, and had the ability to read and speak English.

Survivor participants were recruited using our patient community partners, including Living Beyond Breast Cancers (LBBC), American Cancer Society (ACS), and the Plastic Surgery Foundation (PFS). Potential participants were directed to contact the study team and then were screened and enrolled. Trained research staff conducted the interviews (1 interviewer) and focus groups (2 interviewers) through video conferencing. Both audio and video were recorded, but the video was deleted immediately at the conclusion of the interview, and the audio was sent to a Health Insurance Portability and Accountability Act (HIPAA) compliant third-party service for transcription and de-identification.

We interviewed three types of topical experts: digital/mobile interventions (mHealth) experts, social media or health experts, and health equity experts. To recruit topical experts, the research team, expert advisory group, and CAB engaged their professional networks to share recruitment materials (i.e. recruitment flyers) with possible participants. The participants, if interested, reached out to the research team to confirm eligibility criteria and schedule interviews. We also used snowball sampling from participants, whereby participants could recommend others for interviews. A person was considered an mHealth expert if they had presented or published on the topic, *or* developed or implemented mobile patient-facing health tools, *or* had experience with health-related informatics, *or* had developed or implemented mobile related medical decision-aids. A social media expert was someone with experience managing an organization’s social media account that posts/shares health information *or* had presented or published on health-related social media *or* had a strong social media presence and actively engages about health information. Finally, a health equity expert was someone who had presented or published on equitable dissemination *or* worked on reducing disparities in breast cancer healthcare access *or* had experience disseminating health information to marginalized populations.

### Data analysis

All interviews were transcribed verbatim. A preliminary codebook was created based on deductive (from our adaptation framework) and inductive (emerging from the data) codes related to adaptation. Transcripts were coded using rapid qualitative analysis with NVivo 15 (QSR International Pty Ltd.) software. Rapid qualitative analysis was chosen to organize actionable information efficiently, to inform our adaptations. All transcripts were double coded by two trained coders. Coders reviewed each transcript separately and compared results until inter-rater reliability was reached (kappa > 0.75 and > 95% agreement on each code). Coders discussed any questions regarding the coding with a third team member (VV), who was consulted if consensus was not reached. Codes were organized by the type of code, adaptation suggestion, and section of the website that needed to be modified, based on the adaptation framework and tracked using the FRAME to categorize results. Following data analysis, the research team met to discuss suggestions for the adaptation to BREASTChoice. The ultimate decision was rendered by the full team, with the Principal Investigator of the study making final decisions if consensus was not reached.

## Results

**Fig. 2:**
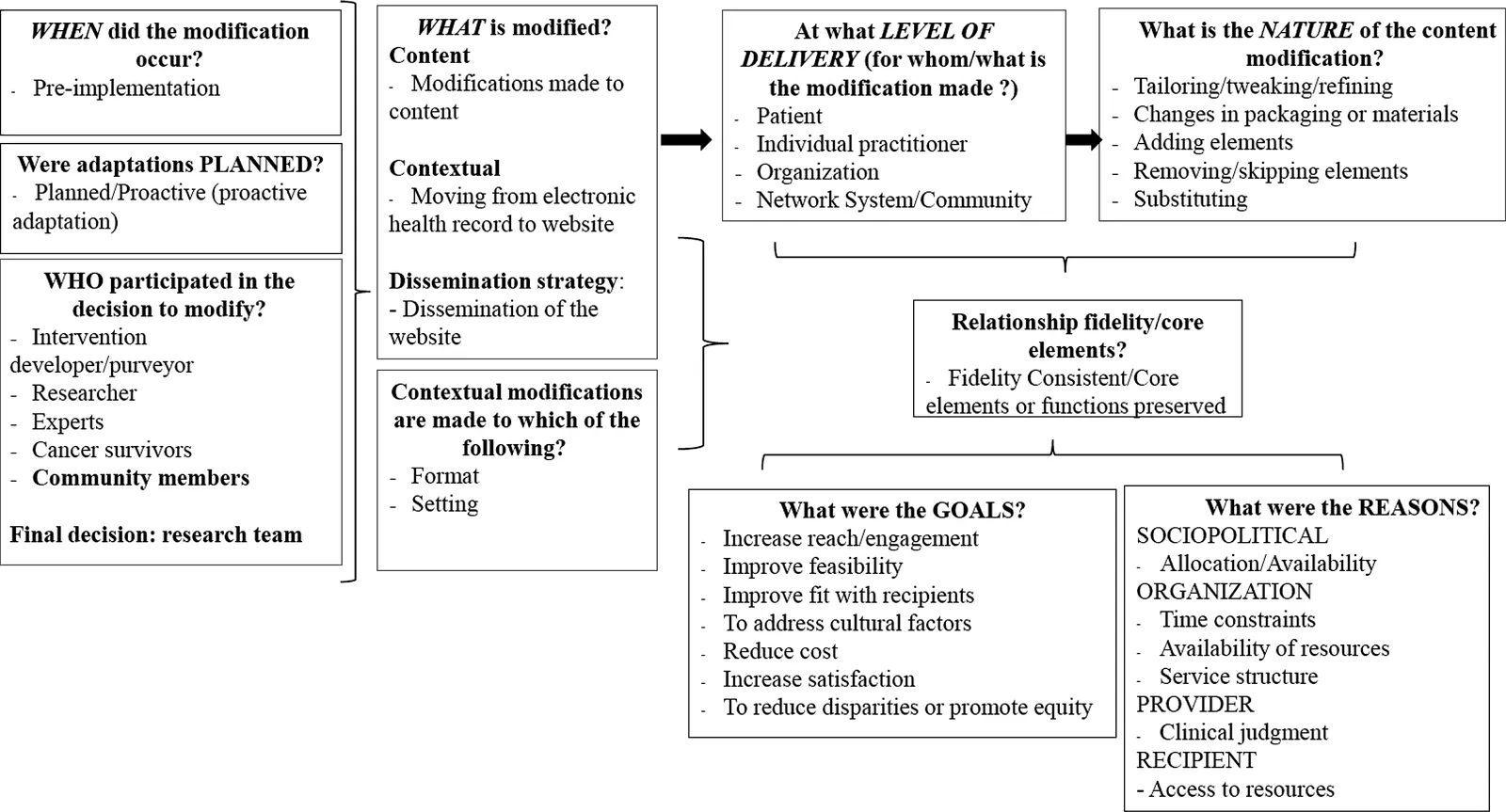
Modified FRAME adapted from Baumann et al. [47] to describe BREASTChoice adaptations

Participants suggested adaptations to the content of BREASTChoice and to the context in how BREASTChoice is used. We used FRAME to categorize and assess each suggested adaptation (see Fig. 2). Below, we describe the content adaptations, context adaptations, adaptations to dissemination strategies suggested by the participants, and the adaptations that were suggested but not implemented.

### Content adaptations

While participants from the breast cancer survivor focus groups praised the content of the website as being informative and helpful, they suggested 72 content modifications. Of these, 7 modifications were about *adding* content (e.g., adding language about nipple sparing mastectomy or nipple tattoos; adding information about costs and insurance coverage), 69 were around *clarifying* information to address *knowledge gaps* (e.g., explaining the difference between silicone and saline implants; improving a description of breast reconstruction or its types; improving a description about the number and length of surgeries, and surgery recovery). Thirteen content modifications were coded as *“other”* and entailed: changing the webpage message to a more neutral tone, changing some colors on the website, adding introductory text to the webpage, or changing wording to be inclusive of those who are thinking about having a mastectomy rather than having already decided (e.g., “If you’re here, you’ve probably been diagnosed with breast cancer and thinking about pursuing a mastectomy to be cancer-free”).

Topical experts generally gave feedback about the interaction of format and content. For example, they suggested decreasing the amount of text, increasing the interactivity of the website, or adding diagrams or figures. As one stated: ‘*I think it’s simple enough to be able to understand*,* but not too much detail…I think patients might get lost in the details…Sometimes I worry that maybe if patients don’t scroll all the way down*,* they may miss an important piece of information*’. Suggestions to address the intersection of content and format in a website included adding key summary sentences and adapting the language to be less academic. A few experts also suggested separating the photos by reconstruction type with more examples: “*That’s what we’re trying to get them to decide. Then within each type have multiple before and after pictures by lots of different skin tones and lots of different body shapes and lots of different breast size and shapes*.”

### Contextual adaptations

Participants from the survivor focus groups showed high enthusiasm about the tool being in a website format outside of the EHR. When reflecting about the benefit of the website, for example, participants shared that having the information on a website allowed them to digest the information at their own pace, to share the information with loved ones or health care team members, so everyone would be aware of the information. For example, one participant described, *“I would like to be able to give somebody the link and have them go on and use it however works for them. I think that there’s something valuable for anyone going through the experience.”* They also shared that the website format allowed them to be empowered to look for other doctors if necessary and to have an informed conversation about their surgeries. As one person shared, *“This would’ve really helped me. I felt like I had to do all the research on my own…I had to literally go out of my region to get my reconstruction done. No plastic surgeon was doing anything but implants in our region.”* Topical experts suggested adding a link to share the different sections of the webpage (i.e., not only the home page), and suggested considering how the webpage would look on different platforms, such as phones and tablets. Experts also mentioned that we could clarify that patients could share the page with their doctors: ‘*I wonder if it might be helpful for the users to understand that it’s easily shareable*,* but what is shared and who it shared with us is under their control.*’

### Adaptations related to dissemination strategy

While the original FRAME only talks about an implementation strategy for clinical settings, here we focus on dissemination strategies, or the processes of sharing information about BREASTChoice through social media and online communities. When asked whether participants would share the website with others, they mentioned that they would share it via Facebook and groups on WhatsApp and Instagram. Interestingly, participants from the focus group mentioned that they would not necessarily share the website via their personal Facebook pages but would rather have it shared via breast cancer groups. For example, one participant commented, *“I may share the link on a Facebook group. You know*,* like maybe a reconstruction group*,* or a triple negative breast cancer group. But I wouldn’t just put it on my newsfeed.”* Another mentioned, *“I would probably copy the link in a group chat*,* maybe with some other ladies. I’d say*,* “Hey*,* you gotta check out this website. If you*,* you know*,* you’re thinking about reconstruction surgery*,* this is the link to the website.”…I’m always trying to share information to other…ladies…that I’ve befriended.”* Experts mentioned reminding users about the sensitive nature of the topic when posting on social media platforms, considering sharing only sections. “*I could see there being content specific content people would be interested in sharing*,* or they just wanna say here’s a whole tool. It is a sensitive topic in some ways.*” Similarly, another expert mentioned “*That might be too private of information that they might not wanna share….* Finally, one expert mentioned that sharing might be useful to prompt engagement: “*I do think something like a big box at the end that says*,* “If this was helpful for you*,* we’d love for you to share it on social media with others*,* and please tag us at”’.*

### Adaptations that were not made

There were some requests for adding content that were not incorporated into the website. For example, participants asked for clarifying information about reconstruction surgery or other breast cancer surgeries, such as how many surgeries might be involved, the estimated time and recovery period for each surgery, non-reconstruction treatments such as lumpectomy. Occasionally, they requested information outside the scope of a reconstruction decision aid, such as resources for talking with children or family members about cancer and reconstruction, or complementary and alternative medicines to support surgery or recovery. Generally, the team decided to not include content that is too patient-specific and not broadly applicable to all (e.g., the specific timeline for recovery is dependent on many factors), or aspects without clear evidence-based guidelines, or topics outside the purpose of the tool. Future work could explore whether it is helpful to have links or resources for more supportive or personalized information that might fall outside the scope of a specific decision in a PtDA.

## Discussion

This paper describes the adaptation of a PtDA from its implementation in clinical settings to dissemination on social media and online communities. We focused on how to accelerate patient access to BREASTChoice, a PtDA about breast reconstruction after mastectomy. We used ADAPT-ITT, a framework that helped delineate how to engage with different voices, to slow the speed and focus on rigorous adaptation. Later, the goal is to increase the speed of reach through broader access from the time to receiving a diagnosis to the time to engaging with evidence-based information to support reconstruction decisions. We are currently piloting the website and evaluating reach and digital usability of BREAST-CHOICE in the next phase of the study.

The underlying assumption of our work is that by sharing the PtDA beyond clinical settings and intentionally and successfully engaging members of typically underserved communities, we could address the limited speed of implementation in health settings and empower patients beyond the boundaries of the healthcare system. This process could improve equity in access to quality care, as patients regardless of baseline access could learn about and discuss evidence-based reconstruction options with their healthcare team.

**Fig. 3:**
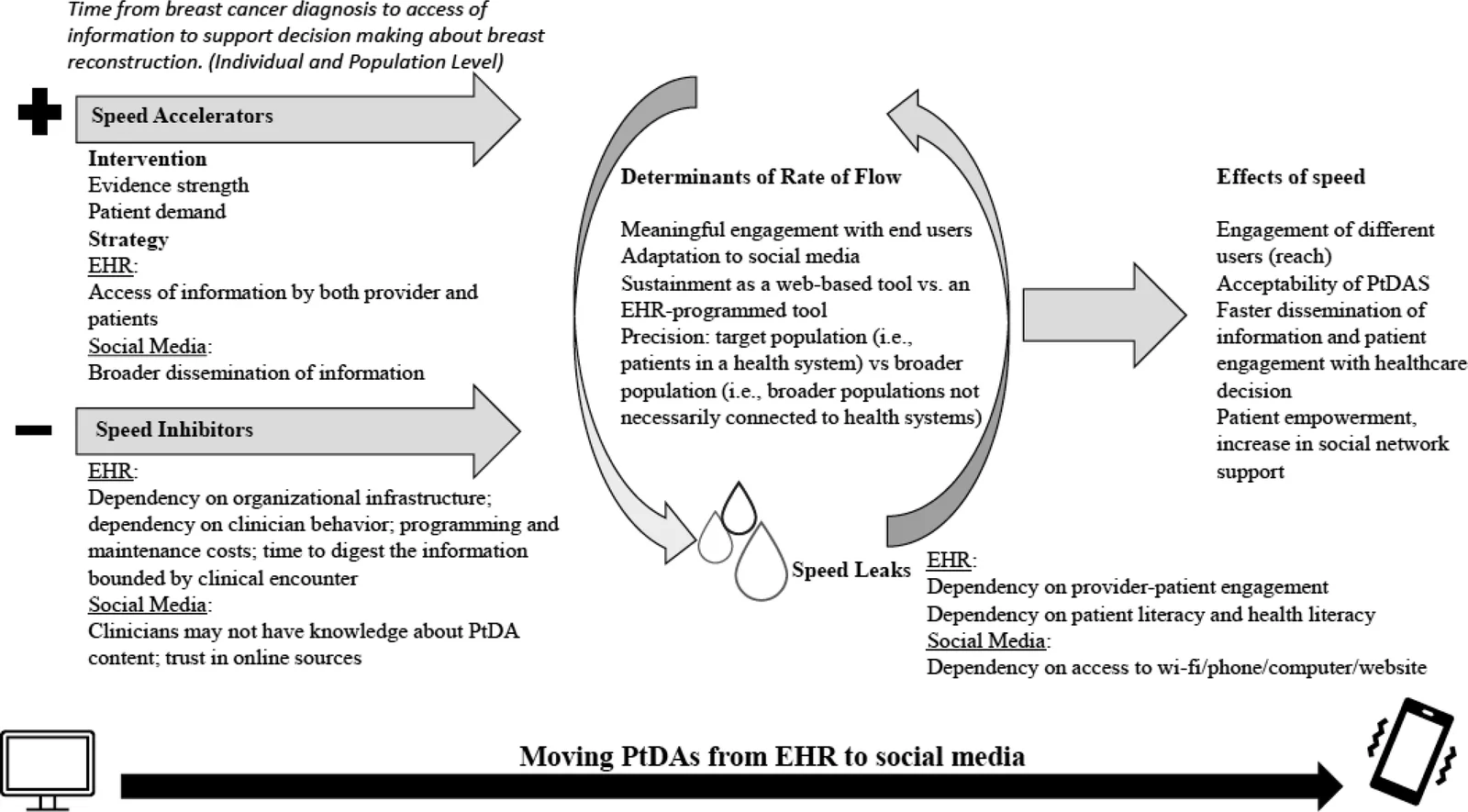
Proposed conceptual framework for measuring determinants of the speed of implementation of an EHR-integrated PtDA, moving it to social media dissemination, adapted from Proctor et al. (2022) [16]

We propose a conceptual framework (Fig. 3) for measuring determinants of the speed of implementation of an EHR-integrated PtDA, moving it to social media dissemination. In this model, we have separated accelerators and inhibitors in terms of the intervention itself and its implementation strategies. We define accelerators as mechanisms of change that reduce the time to impact of an intervention. They strengthen the links between implementation strategies, implementation outcomes, and service and client outcomes. Inhibitors or leaks, on the other hand, are points of failures or breakdowns in the link from the intervention to the outcomes and therefore aspects that can considerably delay the impact.

Table 1 further summarizes leaks, accelerators, and potential outcomes that exist in the contexts of EHR integration and social media dissemination.

**Table 1:** Determinants and Potential Outcomes of Implementation Pace of a Patient Decision Aid, Moving from EHR Integration to Social Media Dissemination. Dimensions Adapted from Proctor et al. (2022) [16]

Dimensions	EHR Implementation	Social Media Dissemination
Accelerators	Evidence strength, patient demand, real-time clinical integration	Broad reach, low cost, user-driven sharing, bypasses organizational barriers
Inhibitors	Organizational infrastructure, clinical workflow, maintenance costs, patient portal use, staff turnover	Digital literacy, trust in online sources, platform dependencies, privacy concerns
Key Leaks	Dependency on clinician + organization + patient portal alignment	May reach unintended or unaffected population; may not reach populations who do not have broadband access; patient may have access to information disconnected from clinical conversation
Rate of Flow	Slower initial reach; deeper integration with care	Faster potential reach; requires targeting for precision
Potential Outcomes		
Reach	Pros: Greater accuracy in reaching the target population Cons: Less reach of those who may not be connected to a healthcare system or healthcare systems supporting use	Pros: potential higher reach of those not connected to the healthcare system, including sharing knowledge with family members and other social networks Cons: Less accuracy in terms of target population but higher reach; potential less equity if those who need the information the most do not have access to social media
Speed (i.e., time from breast cancer diagnosis to first access of information for decision-making)	Potential slower speed	Potential faster speed
Time of engagement with decision aids	Time for conversation potentially bounded by clinical encounter, but with the support of clinician	Time to digest complex information not bounded by clinical encounter

Speed of reach and precision of reach exist in tension. Social media can achieve rapid, broad dissemination, but broad reach may include individuals not facing the target decision – a “leak” that may dilute the impact or introduce noise. In contrast, EHR implementation reaches a narrower population but with high precision: patients are identified at the clinical moment when the decision is relevant. Effective dissemination strategy requires balancing speed against precision, considering not just how many people are reached but whether the right people are reached and whether they are reached at the right moment in their decision-making process [48, 49]. This precision-speed trade-off deserves explicit attention in both implementation and in dissemination sciences.

In terms of accelerators of the intervention, BREASTChoice has strong evidence behind it and patient demand for its use. In terms of accelerators of implementation strategies, implementing it in the EHR allows patients and clinicians to use it together to discuss the decision in real time. On the other hand, the inhibition and potential leaks of the EHR implementation strategy is its dependence on a healthcare organization to integrate tools into the EHR, clinicians to use the EHR and the tool before seeing the patient, and the patient to use the patient portal. The broad and widespread use of social media and online sources for health information, including by people who might not have access to PtDAs in clinical settings is an accelerator of dissemination via social media and online communities. Inhibitors of spread through social media and online communities and potential leaks include limitations in access to social media, trust in online sources, and digital literacy [50–52]. Another potential leak is the possibility that increased reach impacts the patient-clinician relationship. For example, clinicians told us that not all surgeons offer all types of reconstruction, so patients learning about those options via social media could cause tension in the patient-clinician encounter. Clinicians also might not be prepared to address or offer access to PtDAs or might not trust the information in the decision aid. The dissemination of PtDAs through social media could be dependent on access to other infrastructure, such as access to broadband, phones or computers [53, 54]. 

Decisions about breast reconstruction occur within a context that shapes information-seeking and sharing behavior. Survivors may face social judgment about their choices – being perceived as vain for pursuing reconstruction or insufficiently feminine for declining it [55, 56]. Our participants’ preference for sharing BREASTChoice in private breast cancer groups rather than public newsfeeds reflects this context: reconstruction decisions may feel too personal for public disclosure [57]. Social media dissemination strategies must account for privacy and stigma; platforms and sharing mechanisms that feel private or community-bounded may achieve better engagement than public-facing dissemination, depending on the nature of the decision [58]. Understanding how stigma shapes willingness to engage with and share PtDAs is critical for effective dissemination design. The choice of dissemination strategy affects not only speed of reach but also who is reached and at what point in their decision-making journey. Future work should examine how different social media strategies affect the reach, relevance, and impact of PtDA dissemination.

Notwithstanding the leaks, we propose that the speed of moving from the EHR to social media and online communities will ultimately *increase* the reach and acceptability of use of BREASTChoice at the right time in the care pathway, ultimately making its information clinically actionable to improve patients’ health and health care access. The *rate of flow and increase* in speed, however, may depend on the process of adaptation. Engaging end users early in the process of adaptation can improve long term sustainment [59]. We engaged patients, clinicians, and health equity leaders to prevent potential negative outcomes when moving BREASTChoice from the EHR to social media. Such engagement can slow the speed of spread initially but is ethically and scientifically important [60]. Thus, we adapted the PtDA carefully, informed by adaptation frameworks, to ultimately speed up the pace of spread long-term. Our next step is to understand the mechanisms of action [61] of each of these two approaches to reach and examine the impact of the distinct accelerators and inhibitors of the process of patient decision making.

### Limitations

This study is based on a case study of one PtDA, and so the proposed accelerators, inhibitors, and leaks may be unique to BREASTChoice. The framework, while tested across many contexts, was not specifically designed to address the unique considerations of PtDAs, such as digital usability. As of now, the framework is largely descriptive; our next step aims to examine possible mechanisms of action and outcomes of BREASTChoice when disseminated online and through social media. Additionally, participants who engaged in our adaptation process may have been more digitally literate than the broader population of breast cancer survivors, potentially underestimating barriers to social media-based dissemination. The effectiveness of BREASTChoice when accessed through social media – including its impact on decision quality and shared decision-making – remains to be evaluated; this evaluation is the focus of ongoing work. Furthermore, shared decision-making may function differently when patients access PtDAs independently versus within clinical encounters; the tool was designed to support patient-clinician dialogue, and how decision support translates outside that relationship warrants study. Social media dissemination also introduces new sustainability considerations: platform algorithm changes can dramatically affect reach, content moderation policies evolve unpredictably, and ongoing updates are needed as clinical evidence changes. Finally, while social media can achieve broad reach, reaching the right population at the right decision point requires strategic targeting that this adaptation process did not fully address. Nonetheless, this study contributes to implementation and dissemination science and the study of speed and reach by offering important points for discussion when moving an intervention from clinical implementation to broader dissemination.

## Conclusion

We identified determinants and barriers of the pace of implementation of a PtDA, BREASTChoice, in clinical settings, adapting it for dissemination through social media and online communities. Our work indicates the importance of careful adaptation and tracking of steps, slowing the pace of reach at first, to increase the speed and pace of reach by moving the PtDA from one format to another. Our conceptual framework describes how PtDAs can be developed for use within a health system and adapted to spread outside of a health system (i.e., via social media and online communities). The framework can be useful to other PtDA developers or evidence-based interventions that are moving to social media and online platforms.

## Data Availability

The datasets supporting the conclusions of this article will be available in the UNC Dataverse repository at the time of publication.
